# *Notes from the Field:* Tuberculosis Outbreak Linked to a Contaminated Bone Graft Product Used in Spinal Surgery — Delaware, March–June 2021

**DOI:** 10.15585/mmwr.mm7036a4

**Published:** 2021-09-10

**Authors:** Ruoran Li, W. Wyatt Wilson, Noah G. Schwartz, Alfonso C. Hernandez-Romieu, Janet Glowicz, Emily Hanlin, Malikah Taylor, Heather Pelkey, Carol A. Briody, Lija Gireesh, DNP, Mark Eskander, Kenneth Lingenfelter, Sandy P. Althomsons, Rebekah J. Stewart, Rebecca Free, Pallavi Annambhotla, Sridhar V. Basavaraju, Jonathan M. Wortham, Sapna Bamrah Morris, Isaac Benowitz, Maryam B. Haddad, Rick Hong, Marci Drees

**Affiliations:** ^1^Epidemic Intelligence Service, CDC; ^2^Division of Healthcare Quality Promotion, National Center for Emerging and Zoonotic Infectious Diseases, CDC; ^3^Division of Tuberculosis Elimination, National Center for HIV/AIDS, Viral Hepatitis, STD, and TB Prevention, CDC; ^4^Division of Public Health, Delaware Department of Health and Social Services; ^5^ChristianaCare, Wilmington, Delaware.

On May 25, 2021, a Delaware acute care hospital notified the Delaware Division of Public Health (DPH) of seven patients who developed tuberculosis after spinal surgery during March–April 2021. Hospital staff members identified a single common exposure: implantation of bone allograft material (product A) from a single product lot. DPH notified CDC, requested a field investigation, and issued a nationwide call for cases. In collaboration with the Food and Drug Administration, a CDC team was deployed to Delaware on June 2 to investigate the epidemiology of cases and opportunities for transmission and to provide prevention and treatment recommendations. On the same day, another state health department notified CDC about a person who developed tuberculosis after surgery involving the same product A lot, and the manufacturer issued a voluntary nationwide recall (*1*).

Investigators abstracted clinical, laboratory, and imaging data from medical records and interviewed patients. They also assessed potential exposures to *Mycobacterium tuberculosis* related to product A storage, handling, and use during surgery; reprocessing of surgical instruments; and patient care. This investigation was reviewed by CDC and was conducted consistent with applicable federal law and CDC policy.^†^

Twenty-three patients at the hospital underwent surgery that involved the recalled product lot. The median patient age was 66 years (range = 37–80 years). No patient had an immunocompromising condition.^§^ One patient had a history of latent tuberculosis infection and completed treatment in 2003. Nineteen (83%) patients reported new symptoms beginning 2–66 days (median = 19 days) after product implantation (Figure). Fifteen (65%) patients reported constitutional signs and symptoms, including fever, chills, night sweats, weight loss, fatigue, and loss of appetite; 16 (70%) had redness, pain, or drainage at the surgical site; four (17%) experienced neurologic symptoms, including paresthesia and dysphagia; and seven (30%) experienced pulmonary symptoms, including cough and shortness of breath. Four (17%) patients were asymptomatic. Sixteen (70%) required hospital readmission 23–84 days after product implantation (median = 52 days). Twelve (52%) underwent additional surgical procedures to manage complications of infection. One patient died at home 3 weeks after product implantation, which was 2 months before the product recall.

**FIGURE:**
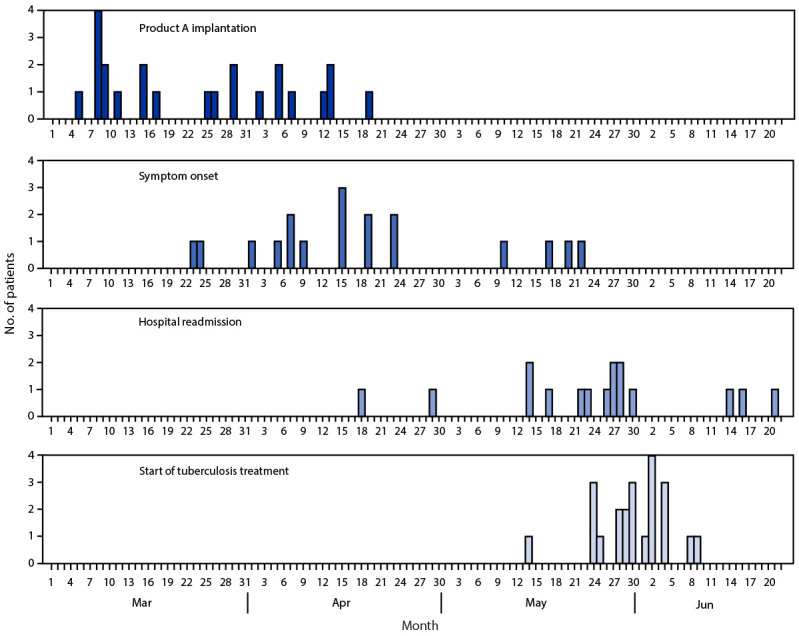
Time line of product implantation, symptom onset,* hospital readmission,^†^ and start of tuberculosis treatment^§^ in patients exposed to product A^¶^ (N = 23) — Delaware, March–June 2021 * Excludes one patient with insidious onset of weight loss. ^†^ One patient was readmitted 4 days after product implantation with a surgical site infection caused by nonmycobacterial species; this readmission was excluded from the figure. The patient subsequently developed worsening lumbar back pain and was readmitted with a tuberculous abscess; this subsequent symptom onset and readmission were included in the figure. ^§^ All 22 living patients have started tuberculosis treatment. One patient died 3 weeks after product implantation, before the tuberculosis outbreak was recognized. ^¶^ Numbers of patients in each category are as follows: product A implantation = 23 patients; symptom onset = 18; readmission = 16; start of tuberculosis treatment = 22.

As of June 25, 19 (83%) of the 23 patients had laboratory or imaging evidence of tuberculosis in the spine or chest. Among 19 patients who had received microbiologic testing of a vertebral, paraspinal soft tissue, or sputum specimen, 15 (79%) had a positive acid-fast bacilli smear, *M. tuberculosis* nucleic acid amplification test, or culture. Among 21 patients who had received spinal imaging, 19 (90%) had findings consistent with infection, including abscesses (17, 81%) and osteomyelitis or discitis (eight, 38%). Among 21 patients with sputum testing or chest imaging, six (28%) demonstrated evidence of pulmonary tuberculosis, suggesting bloodborne dissemination of *M. tuberculosis*. Isolates cultured from specimens from three patient specimens were susceptible to all first-line medications and shared a genotype not previously identified in the United States; drug-susceptibility testing and genotyping of subsequent isolates were pending. All 22 living patients began standard four-drug treatment for drug-susceptible tuberculosis 41–91 days after product implantation (median = 69 days).

Product A was shipped frozen and was opened inside the operating room. Health care personnel could have been exposed to *M. tuberculosis* during product A implantation and subsequent procedures involving suctioning, drilling, wound irrigation, and decontamination of cannulated instruments. In addition, health care personnel and patients could have been exposed to *M. tuberculosis* from patients who had received implantations with product A and subsequently developed draining tuberculous lesions or pulmonary tuberculosis (*2**–**4*). As of June 24, the hospital had identified 152 health care personnel and seven patients who were exposed to recipients of product A who had tuberculous abscesses or pulmonary tuberculosis; investigations are ongoing to identify additional exposures.

This investigation found high attack rates of spinal and disseminated tuberculosis after surgical implantation of product A and multiple opportunities for *M. tuberculosis* exposure related to surgery and patient care. On June 4, CDC recommended that all patients nationwide who had undergone surgery involving the affected product A lot be immediately assessed and begin the four-drug treatment for tuberculosis disease, even if they were asymptomatic. On June 8, CDC recommended evaluation of contacts in health care settings, including risk assessment, symptom screening, and an interferon-gamma release assay or tuberculin skin test (*3,4)*.

*M. tuberculosis* transmission via bone graft was last described in 1953 (*5*). The hospital’s rapid detection of this unusual cluster triggered a multistate investigation resulting in sequestration of all unused units of the contaminated product, identification of all patients who underwent surgical procedures with the contaminated product lot, and initiation of tuberculosis treatment by all living patients. Public health authorities and health care facilities should continue efforts to identify and evaluate all exposed contacts and identify opportunities to prevent *M. tuberculosis* exposures from contaminated tissues or other products.

## Footnotes

* These authors contributed equally to this report.

† 45 C.F.R. part 46, 21 C.F.R. part 56; 42 U.S.C. Sect. 241(d); 5 U.S.C. Sect. 552a; 44 U.S.C. Sect. 3501 et seq.
